# Development of an outcome indicator framework for a universal health visiting programme using routinely collected data

**DOI:** 10.1186/s12913-024-11178-7

**Published:** 2024-06-14

**Authors:** Margaret Horne, Louise Marryat, D. Helen Corby, Lawrence Doi, Ruth Astbury, Ruth Jepson, Kathleen Morrison, Rachael Wood

**Affiliations:** 1 Salvesen Mindroom Research Centre, Centre for Clinical Brain Sciences, University of Edinburgh, Edinburgh, UK; 2 School of Health Sciences, University of Dundee, Dundee, UK; 3 Scottish Collaboration for Public Health Research and Policy, School of Health in Social Science, University of Edinburgh, Edinburgh, UK; 4 School of Health and Life Sciences, University of West of Scotland, Paisley, UK; 5 Public Health Scotland, Edinburgh, UK

**Keywords:** Home visiting, Health visiting, Public health nurses, Administrative data, Framework, Methods

## Abstract

**Background:**

Universal health visiting has been a cornerstone of preventative healthcare for children in the United Kingdom (UK) for over 100 years. In 2016, Scotland introduced a new Universal Health Visiting Pathway (UHVP), involving a greater number of contacts with a particular emphasis on the first year, visits within the home setting, and rigorous developmental assessment conducted by a qualified Health Visitor. To evaluate the UHVP, an outcome indicator framework was developed using routine administrative data. This paper sets out the development of these indicators.

**Methods:**

A logic model was produced with stakeholders to define the group of outcomes, before further refining and aligning of the measures through discussions with stakeholders and inspection of data. Power calculations were carried out and initial data described for the chosen indicators.

**Results:**

Eighteen indicators were selected across eight outcome areas: parental smoking, breastfeeding, immunisations, dental health, developmental concerns, obesity, accidents and injuries, and child protection interventions. Data quality was mixed. Coverage of reviews was high; over 90% of children received key reviews. Individual item completion was more variable: 92.2% had breastfeeding data at 6–8 weeks, whilst 63.2% had BMI recorded at 27–30 months. Prevalence also varied greatly, from 1.3% of children’s names being on the Child Protection register for over six months by age three, to 93.6% having received all immunisations by age two.

**Conclusions:**

Home visiting services play a key role in ensuring children and families have the right support to enable the best start in life. As these programmes evolve, it is crucial to understand whether changes lead to improvements in child outcomes. This paper describes a set of indicators using routinely-collected data, lessening additional burden on participants, and reducing response bias which may be apparent in other forms of evaluation. Further research is needed to explore the transferability of this indicator framework to other settings.

**Supplementary Information:**

The online version contains supplementary material available at 10.1186/s12913-024-11178-7.

## Introduction

A healthy childhood sets the stage for health across the life course [1]. Universal health visiting provision has been a cornerstone of preventative healthcare for children in the United Kingdom (UK) for more than 100 years [2]. In that time many changes have taken place, although the foundations of a home visiting programme covering a wide array of child and parental health assessment and support has continued. Health visiting, as with all health policy in Scotland, has been devolved from UK policy since 1999, leading to changes which are specific to Scotland. In 2003, the Health for All Children, version 4 (Hall 4) was published with specific recommendations that a core number of contacts should be established for all children (the ‘Core’ group), with additional visits beyond the 6–8 week check being provided for those deemed to be in need of additional support. The majority of visits/contacts were to be carried out by a range of health professionals [3]. Research on this approach demonstrated that the identification of additional support needs at six to eight weeks missed substantial proportions of children who later displayed language problems (43% of those with language difficulties at 30 months being in the ‘Core’ group at 6–8 weeks), or social, emotional and behavioural difficulties (64% having previously been allocated to the ‘Core’ group) [4]. This led to a revision of the Hall 4 policy in Scotland, which reintroduced a universal review of the child at 24–30 months [5].

Over the subsequent years, further evidence indicated inconsistencies in the delivery of health visiting [6], whilst the introduction of a Named Person service for young people, where a named person is the single point of contact if a child or their parents want information or advice, led to the health visitor role being increasingly important as the named person for children prior to starting school, usually at age 5 years [7].

**Fig. 1:**
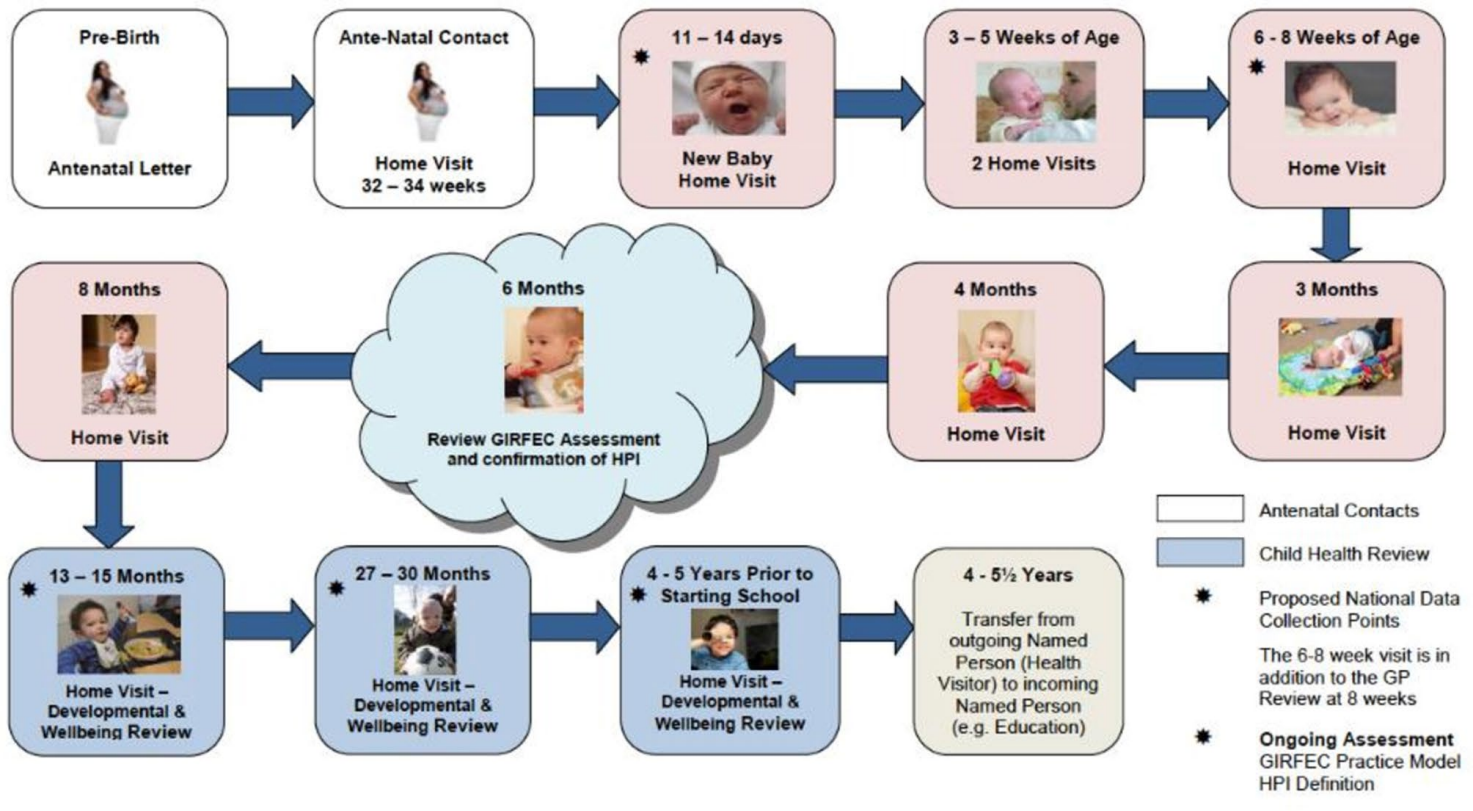
Universal Health Visiting Pathway timeline (produced and published by the Scottish Government [8])

In 2015, this led to the publication of the new Universal Health Visiting Pathway (UHVP) policy [8], which re-emphasised both the role of the health visitor in home visiting and the importance of regular universal home visits throughout the preschool years with a particular focus on the first year of life (Fig. 1).

Additionally, the UHVP placed greater importance on visits being conducted in the home setting, involving both parents where applicable, and using robust developmental assessments [8]. The UHVP was intended to be implemented across Scotland for children born on or after 1 April 2016. In reality, however, implementation varied across health boards, with the earliest point of implementation occurring for children born in April 2015, and the latest for children born in October 2019.

Monitoring child health outcomes is of primary concern to policymakers in many high-income countries, including Scotland.This has led to a wealth of administrative data being collected about child health, particularly in the early years. Among administrative datasets available, Child Health Surveillance Programmes (CHSP) aim ‘to prevent disease, detect physical and developmental abnormalities, and promote optimum health and development’ [9]. In many high-income countries these surveillance programmes have now been running for many years, making them an ideal source of data for capturing trends over time, and providing opportunities to use quasi-experimental techniques to explore the impacts of national policy interventions. The proposed indicator framework makes use of the Scottish CHSP, collected primarily by health visitors, supplemented by other routine data sources where necessary.

Although other indicator frameworks of child health have been developed [10], these frameworks contain limited application for the Scottish context, due both to cultural differences in service delivery, and, more importantly, a tendency to focus on programme-based data collection, rather than universal health surveillance-based indicators. Ben-Arieh [11] notes that administrative data are likely to be the best option for developing new sets of children’s wellbeing indicators, due to both the expense of alternative approaches, such as surveys, and the abundant availability of administrative data. This is particularly true when looking at outcomes across a whole population, as is the case in this current study, which uses administrative data collected as part of the CHSP, alongside other routine data sources such as hospital admissions, to explore a range of outcomes which can potentially be attributed to the actions of health visitors.

The UHVP represents a significant financial commitment from the Scottish Government of £40 million to increase health visiting staff numbers to deliver the pathway. Public bodies require evidence that changes being made as the result of such investment are benefitting the target population (in this case improving outcomes for children and families) in order to justify continuing expenditure. To evaluate the impact of the UHVP on a range of outcomes, the Scottish Government therefore commissioned a research consortium to robustly evaluate the implementation and outcomes for the programme. The aim of this paper is to outline the process of developing an outcome indicator framework, specifically including outcomes that could be measured using routinely-collected data as part of this UHVP evaluation. The paper will document the process and decisions that led to the creation of a set of administrative data indicators, what those indicators comprise, and the baseline data from those indicators.

## Methods

### Setting

This study was undertaken in Scotland, where there are approximately 55,000 births per year. As part of the National Health Service, health visiting is a free at point of use service provided to all new parents in the UK. As part of this, everyone is given a universal identifier (Community Health Index (CHI) number) which allows their health data to be linked over time. Set data are collected as part of the health visitor reviews each time they visit. A subset of these data are collated at a national level and can be made available to research. This evaluation sought to utilise these routine data at an aggregate level. Administrative data should, in theory, therefore be available for all children born in Scotland, or who subsequently moved to Scotland and registered with a GP. In reality, not all parents will take up the offer of an appointment, and/or may refuse to answer individual questions: for this reason, not all children will have data available for every review/variable. Missingness is discussed in the results section.

### Methods

Before beginning the evaluation of the UHVP, an evaluability assessment was conducted [12]. As part of the assessment a theory of change was produced [13]: this involved a series of workshops with stakeholders to explore the UHVP implementation processes and to define their anticipated outcomes. The criteria for outcomes were: (1) outcomes that relate to the child and family, and (2) outcomes which could feasibly be influenced by Health Visitors through the pathway. This formed the basis of an initial logic model [12] to visually explain the pathways from the activities (e.g. home visits) to the selected outcomes (e.g. health childhood development and early identification of problems). Following the implementation of the UHVP, this logic model was revisited in a further series of workshops, bringing together the research team with 31 health visitors and other health professionals and managers, policy-makers, and third-sector organisations; resulting in the final logic model for the evaluation of the UHVP [14]. The research team then mapped outcomes identified within the logic model to the most appropriate methods for assessing them (sometimes comprising more than one method). This resulted in four distinct workstreams of qualitative evaluation, case notes review, surveys and routine data. The full methodology can be found in the UHVP Protocol paper [15].

Four broad outcome groupings from the logic model were allocated to the routine data analysis stream of the evaluation (Table 1). This paper focuses on the range of outcomes within these grouping which were:

i) improved health behaviours within families;
ii) improved child development and school readiness;
iii) improved health outcomes for children;
iv) improved child safety and protection.

Administrative data were then sought which were nationally available at both the pre- and post-UHVP implementation stages, and addressed the outcome groupings. To confirm face validity and feasibility, a provisional list of outcome indicators was shared with wider members of the UHVP evaluation team (which included academics from health visiting, community paediatrics and social work and a senior Scottish Government analyst working on child protection data) for comment.

Further discussion on this provisional list was undertaken with Public Health Scotland and Scottish Government analysts to obtain indicative background information on: the quality of the data source to be used for each measure; and how common the chosen outcomes were. This led to certain provisional measures being refined or dropped. Further review by the Research Advisory Group for the project (which included third-sector colleagues, health professionals and policy-makers) led to the inclusion of two additional measures on looked after children, supplementing those already included on children on the child protection register.

Preliminary aggregate data (by Health Board) were sought to assess quality of data sources and power calculations were performed. This is important for any future evaluation to ascertain if any change could be reasonably identified in the data. Information was used to estimate the statistical power to detect a 5%, 10%, and 20% relative change in each of the outcomes of interest in the Scottish context (Supplementary Table 1). As there was no clear a priori information available on the expected impact of the UHVP on the chosen outcome indicators, estimating the power to detect a 5%, 10%, and 20% relative change gave a useful indication of power to detect relatively modest, but potentially feasible impacts that would represent meaningful improvements at the population level. All methods carried out in the study were performed in accordance with relevant guidelines and regulations, such as the Public Health Scotland Statistical Disclosure Protocol [16] .

All data analysed were secondary data held by the Scottish Government and Public Health Scotland, respectively. These aggregate data were made available to the research team by the respective organisations through Scottish Government and Public Health Scotland colleagues working with us as part of the evaluation. Details on how others can access these data are available at the end on the paper.

The evaluation received ethical approval from the School of Health in Social Science Research Ethics Committee, University of Edinburgh.

## Results

Within the broad outcome groupings, the final outcome indicator framework comprised 18 indicators across eight core outcome areas: parental smoking, breastfeeding, immunisations, dental health, developmental concerns, obesity, accidents and injuries, and child protection interventions (Table 1). One element (‘school readiness’) was not able to be assessed within the routinely-collected data.

**Table 1:** Final routine data outcomes alongside the logic model item

Logic Model Item	Specific Outcome indicators
1. Improved health behaviours within families (e.g., smoke-free homes, breast feeding, weaning and early diet, oral health)	Parental smoking 1a. Primary carer current smoker at 27–30months 1b. Child exposed to second hand smoke at 27–30months Infant feeding 1c. Exclusive breast milk feeding at 6–8weeks 1d. Any breast milk feeding at 6–8weeks Immunisations 1e. Complete coverage of universal primary and end infancy immunisations by second birthday Dental care 1 f. Any attendance at dentist by second birthday
2. Improved child development and school readiness	Developmental concerns 2a. Any developmental concern at 27–30months 2b. Any concern about speech, language and communication development at 27–30months 2c. Any concern about social and emotional development at 27–30months
3. Improved health outcomes for children (e.g., healthy child weight, reduced hospital admissions for serious injuries)	Child BMI 3a. Child at risk of overweight or obesity (BMI ≥ 85th centile) at 27–30 months 3b. Child clinically obese (BMI ≥ 98th centile) at 27–30months Unintentional injuries 3c. Any hospital admission for unintentional injury by third birthday 3d. Any hospital admission for unintentional poisoning, burn or scald by third birthday 3e. Any hospital admission for unintentional long bone fracture or head injury by third birthday
4. Improved child safety and protection	Child protection interventions 4a. Placed on child protection register at any point between birth and third birthday 4b. Placed on child protection register for ≥ 6months between birth and third birthday 4c. ‘Looked After Child’ status at any point between birth and third birthday 4d. ‘Looked After Child’ status for ≥ 6months between birth and third birthday

BMI, body mass index; SIMD, Scottish Index of Multiple Deprivation

Data quality was mixed: coverage of health visitor reviews, in which data are collected for the CHSP, was high, with over 90% of children receiving their 6–8 week review, and over 90% receiving their 27–30 month review. Individual item completion within reviews was far more variable, ranging from 63.2% for child BMI (used to calculate overweight and obesity) at 27–30 months, to 92.2% for breastfeeding outcomes at 6–8 weeks. Completeness of other (non-CHSP) sources was assumed, e.g. hospital data, whereby a lack of a record of hospital admission is assumed to mean there was no admission. Accuracy of data recording is difficult to quantify for routinely-collected data, however analysis from Public Health Scotland indicates that the accuracy of diagnostic coding in Hospital Admissions data, for example, is high [17].

Alongside coverage, utility of the data also depends on the prevalence within the population. Although the majority of indicators chosen fall in the middle range of prevalence, eight indicators, relating to three outcomes, demonstrated extreme high or low prevalence. Overall, 93.6% children had received all immunisations by age 2 years. Conversely, levels of accidents and injuries were found to be very low, with 3.4% having *any* hospital admission for unintentional injury by age three, and child protection indicators were equally low, from 1.3% children having been on the Child Protection register for more than six months between birth and the child’s third birthday, to 2.7% being placed on the child protection register during the same period (Supplemental Table 2.

Corresponding power calculations, which were undertaken based on indicative data prior to the evaluation commencing, demonstrate the impact of these prevalence rates on the power to detect change. Although there is adequate statistical power to detect modest levels of impact (20% relative change or less) of the UHVP on the majority of outcome measures, this is not necessarily the case with indicators with very low prevalence i.e. unintentional injuries and child protection measures. These also require follow-up to a child’s 3rd birthday, reducing the number of children that can be included in the exposed group in these data. This means that our power to detect modest impact on these outcomes is relatively low. Consequently, only more substantial impacts/differences between unexposed and exposed groups will be identified as statistically ‘significant’. For example, we will have estimated 61% power to detect a 20% change in the proportion of children admitted for unintentional poisoning, burns or scalds by their 3rd birthday as statistically significant (at the 1% significance level). This is still a feasible level of impact; hence all agreed outcome indicators are likely to be informative to some degree.

## Discussion

The final indicator framework comprised eighteen indicators across eight core outcome areas and four broad groupings. The eight outcome areas were: parental smoking, breastfeeding, immunisations, dental health, developmental concerns, obesity, accidents and injuries, and child protection interventions. These were felt to be key to child health, as well as being outcomes that health visitors were able to influence. Many of these are central to policy priorities, not only in Scotland, but across the world, as they form risk indicators for health outcomes across the life course: these included exposure to second-hand smoke, being breastfed, receiving childhood immunisations, and being overweight or obese [18–21].

Dental attendance was included as a pathway to improved dental health: health visitors discuss dental health, registration and attendance with parents in infancy and beyond. Dental health among children in Scotland is particularly poor, especially among children living in more deprived areas and those with Looked After Status [22]. This is an area where health visitors have the potential to improve outcomes through encouraging toothbrushing and dentist attendance. Developmental data are important for two reasons: first, health visitors work with parents to encourage activities that aid child development, e.g. reading, singing and play; and second, health visitors are key to identifying delayed development in early childhood as well as advising parents of and referring to appropriate services, whether that be diagnostic services, speech and language therapy, or early access to free preschool places [4]. Accident and injury data attempt to capture accidents and injuries in the early years which are largely preventable, such as burns, scalds and head injuries, the majority of which happen in the home setting [23]. Health visitors work with parents to put in place preventative measures such as locks on cupboards and stairgates, as well as discussing supervision of children. Finally, health visitors play a key role in identifying, alongside social workers, where families are struggling to cope. For this reason a range of child protection indicators were included in the framework. On the advice of specialists we consulted in this field, including academic social workers, measures of numbers of child protection registrations and children with Looked After Child status, as well as the length of time their names were spent either on the register or they were in care, were included. This is because it was felt that through seeing children more regularly in the early years, health visitors might make more referrals to social work with regards to child protection concerns, resulting in increases to these figures; if intervention occurred at an earlier stage, children and families should receive appropriate support in a timely manner, and thus may spend less time with their names on the child protection register or in the care of a Local Authority .

It is notable though that indicators were only captured for fields in which national administrative data were collected. This meant that some outcomes which were deemed important to measure, such as quality of parent-child relationship or parental efficacy, could not be captured due to a lack of available quantitative data. Attachment behaviour is not currently captured in national data and it is debatable the extent to which this could be quantitatively measured at this scale. Attachment is robustly measured in an experimental setting through either observation of parent and child, or through a story-play task, usually conducted by a specially trained psychologist [24]. This is not practical at a population level. This is in contrast to previous child health indicator frameworks which used a wider range of data collected specifically to evaluate the programme and are thus able to capture some of these ‘softer’ measures [25]. As part of a national health service, resources are limited, and focus therefore is, understandably, directed towards provision of care, rather than data collection per se. Whilst Public Health Scotland frequently revisit which data are collected, it is not currently possible to collect and collate qualitative data at a national level.

Although the framework has been defined by Scottish health and social care professionals, policy-makers and researchers, based on available Scottish administrative data, it has potential to be adapted and implemented for evaluation of child health service provision, and home visiting programmes, internationally. Previous research has demonstrated similar indicators of interests, however the challenge to date has been around access to consistent and high quality data [26]. As countries have increasingly sophisticated data infrastructure programmes, the ability to monitor and assess progress towards enhancing children’s experiences and outcomes will only improve.

In addition, whilst the suggested framework covers many key components of child health and development, some of these factors, such as accidental injuries and child protection interventions, are (thankfully) relatively rare. Even in a population of more than 50,000 births per year, this causes problems in the power to demonstrate smaller changes following the implementation of an intervention such as the UHVP. By contrast, a ceiling effect may be present for high-prevalence outcomes such as childhood immunisation, where very few children do not receive all immunisations. Of course, this has the potential to change if the framework is used in different cultural contexts and highlights the importance of indicators being assessed in relation to the population in question before use.

Although data coverage is high, on the whole, this was not the case with BMI measurements. This varied substantially by health board. Levels of overweight and obesity were high: 40.3% of children who were measured were overweight or obese at 27–30 months, compared with 22.8% at age 4–5 years [27]. The researchers hypothesise that this may be related to selective weighing of apparently heavier children, as well as the use of the WHO growth standard, which is based on ‘optimal’ breastfed babies (i.e. mothers who were non-smokers, no health, environmental or economic constraints on growth; absence of significant morbidity; gestational age 259–294 days; and single term birth), rather than the UK90 standard [28, 29]. There is therefore a risk that any increase in improvements in coverage of this measurement will result in artificial improvements in this indicator, and this needs to be closely monitored.

### Strengths and limitations

The strengths of this study are that it comprises a robust approach to development of a suite of measures which (a) reflect important aspects of the health, development, and wellbeing of pre-school children, (b) may be influenced by child health/home visiting programmes, and (c) are likely to be measurable using routinely available administrative data in high-income countries. The indicators were informed by a wide range of discussions with health professionals, policy-makers, academics and third-sector organisations, and cover a wide range of domains deemed to be important to early child health. Overall, data quality was high, with the exception of height and weight data, where a large proportion of data were missing, and high levels of overweight/obesity in the available data indicate that this might not be at random.

Indicators were further limited by the availability of national data, resulting in some indicators of interest, e.g. attachment, not being able to be included in the indicator framework. Additionally, some outcomes had extremely low or high prevalence, which may limit their use. The indicator framework would need to be assessed for cultural appropriateness before transferring to other settings.

## Conclusions

Good health in childhood is associated with more positive outcomes in adulthood [1]. In high-income countries, such as Scotland, home visiting services, such as that undertaken by health visitors, plays a key role in ensuring that children and their families have the right support to enable the best start in life. As home visiting programmes evolve, it is crucial to understand whether changes lead to improvements in child outcomes. This paper sets out the process for developing a set of indicators using routinely-collected data, lessening additional burden on participants, and reducing response bias which may be apparent in other forms of evaluation. The resultant framework contained 18 indicators across 8 key outcomes and four broad groupings, allowing robust identification of trends and changes across time. Further research is needed to explore the transferability of this indicator framework to other settings.

### Electronic supplementary material

Below is the link to the electronic supplementary material.

Supplementary Material 1

Supplementary Material 2

## Data Availability

All data analysed were secondary data held by the Scottish Government and Public Health Scotland, respectively. These aggregate data were made available to us by the respective organisations through Scottish Government and Public Health Scotland colleagues working with us as part of the evaluation. Data are available to other researchers through these organisations: some data at an aggregate level are publicly available (e.g. https://www.opendata.nhs.scot/), whilst others might need to be obtained through FOI requests (e.g. https://www.nss.nhs.scot/how-nss-works/freedom-of-information/) Individual level data can be obtained through the Electronic Data and Innovation Service (EDRiS): https://publichealthscotland.scot/services/data-research-and-innovation-services/electronic-data-research-and-innovation-service-edris/overview/what-is-edris/.
