# Supplementary material for: Development of an outcome indicator framework for a universal health visiting programme using routinely collected data
Source: BMC Health Serv Res. 2024 Jun 14;24:728. doi: 10.1186/s12913-024-11178-7 (PMC11177436; doi:10.1186/s12913-024-11178-7)
Supplement: Supplementary file 1 — Supplementary Material 1 [file 12913_2024_11178_MOESM1_ESM.docx]

**Supplementary Table 1: Estimated statistical power to detect 5%, 10%, and 20% relative change in the outcomes of interest for children exposed (compared to unexposed) to the UHVP**

| **Outcome** | **Estimated number unexposed^1^** | **Estimated number (%) of unexposed with valid data** | **Estimated number (%) of unexposed with valid data who have outcome of interest** | **Estimated total number exposed^2^** | **Estimated number (%) of exposed with valid data** | **Power to detect stated relative % change in proportion with outcome of interest at (two-sided) 1% significance level** | | |
| --- | --- | --- | --- | --- | --- | --- | --- | --- |
|  |  |  |  |  |  | **5%** | **10%** | **20%** |
| Primary carer current smoker at 27-30m | 331,541 | 290,838  (87.7%) | 46,703  (16.5%) | 37,875 | 33,200  (87.7%) | 62.5% | >99.5% | >99.5% |
| Child exposed to second hand smoke at 27-30m | 331,541 | 289,135  (87.2%) | 23,500  (8.4%) | 37,875 | 33,000  (87.2%) | 27.0% | 91% | >99.5% |
| Exclusive breast milk feeding at 6-8w | 331,541 | 296,956  (89.6%) | 82,001  (27.6%) | 143,230 | 128,289  (89.6%) | >99.5% | >99.5% | >99.5% |
| Any breast milk feeding at 6-8w | 331,541 | 296,956  (89.6%) | 114,018  (38.4%) | 143,230 | 128,289  (89.6%) | >99.5% | >99.5% | >99.5% |
| Complete coverage of universal primary and end infancy immunisations by 2^nd^ birthday^3^ | 331,541 | 331,541  (100%) | 298,387  (90%) | 72,076 | 72,076  (100%) | >99.5% | >99.5% | n/a |
| Any attendance at dentist by 2^nd^ birthday^5^ | 331,541 | 331,541  (100%) | 220,873  (68.6%) | 72,076 | 72,076  (100%) | >99.5% | >99.5% | >99.5% |
| Any developmental concern at 27-30m | 331,541 | 299,100  (90.2%) | 46,183  (15.4%) | 37,875 | 34,169  (90.2%) | 60% | 99% | >99.5% |
| Any concern about speech, language and communication development at 27-30m | 331,541 | 299,100  (90.2%) | 32,086  (10.7%) | 37,875 | 34,169  (90.2%) | 38% | 98% | >99.5% |
| Any concern about social and emotional development at 27-30m^8^ | 331,541 | 299,100  (90.2%) | 17,946  (6.0%) | 37,875 | 34,169  (90.2%) | 18% | 79% | >99.5% |
| **Outcome** | **Estimated number unexposed^1^** | **Estimated number (%) of unexposed with valid data** | **Estimated number (%) of unexposed with valid data who have outcome of interest** | **Estimated total number exposed^2^** | **Estimated number (%) of exposed with valid data** | **Power to detect stated relative % change in proportion with outcome of interest at (two-sided) 1% significance level** | | |
|  |  |  |  |  |  | **5%** | **10%** | **20%** |
| Child at risk of overweight or obesity (BMI ≥85^th^ centile) at 27-30 months | 331,541 | 204,612  (61.7%) | 83,666  (40.9%) | 37,875 | 23,375  (61.7%) | 97% | >99.5% | >99.5% |
| Child clinically obese (BMI ≥98^th^ centile) at 27-30 months | 331,541 | 204,612  (61.7%) | 24,709  (12.1%) | 37,875 | 23,375  (61.7%) | 29% | 94% | >99.5% |
| Any hospital admission for unintentional injury by 3rd birthday^11^ | 331,541 | 331,541  (100%) | 11,287  (3.4%) | 37,875 | 37,875  (100%) | 10% | 52% | >99.5% |
| Any hospital admission for unintentional poisoning burn or scald by 3^rd^ birthday^12^ | 331,541 | 331,541  (100%) | 3,183  (0.96%) | 37,875 | 37,875  (100%) | <3% | 12% | 61% |
| Any hospital admission for unintentional long bone fracture or head injury by 3^rd^ birthday^13^ | 331,541 | 331,541  (100%) | 5,958  (1.8%) | 37,875 | 37,875  (100%) | 5% | 25% | 90% |
| Placed on child protection register at any point between birth and 3^rd^ birthday | 331,541 | 331,541  (100%) | 10,952  (3.3%) | 37,875 | 37,875  (100%) | 10% | 51% | >99.5% |
| Placed on child protection register for ≥6 months between birth and 3^rd^ birthday | 331,541 | 331,541  (100%) | 5,058  (1.5%) | 37,875 | 37,875  (100%) | 4% | 20% | 85% |

**Notes**

|  | Adequate (≥90%) power to detect stated level of impact on this outcome |
| --- | --- |

* Only provided for outcomes with <90% to detect a 20% relative change

^1^ Those children who did not receive any part of the new UHVP pathway (receiving instead the previous Health Visiting reviews)

^2^ Those children who were born on or after the date on which the UHVP was fully implemented in the health board in which the child was born.
