# Supplementary material for: Development of an outcome indicator framework for a universal health visiting programme using routinely collected data
Source: BMC Health Serv Res. 2024 Jun 14;24:728. doi: 10.1186/s12913-024-11178-7 (PMC11177436; doi:10.1186/s12913-024-11178-7)
Supplement: Supplementary file 2 — Supplementary Material 2 [file 12913_2024_11178_MOESM2_ESM.docx]

**Supplemental Table 2 Background data to child health indicators – data based on all children born in Scotland between 1 January 2011 and 31 March 2016**

| **Outcome** | **Indicator** | **Data source** | **Total number of infants in cohort^1^** | **Mean number of infants per quarter^2^** | **Children receiving review (mean n.)** | **Review coverage (mean %)** | **Review records with valid data on specified outcome (mean n.)** | **Overall cohort with valid data (mean %)** | **Children recorded as having the outcome of interest (mean n.)** | **Children with valid data who have outcome of interest (mean %)** |
| --- | --- | --- | --- | --- | --- | --- | --- | --- | --- | --- |
| Parental smoking | Primary carer current smoker at 27–30months | Child Health Surveillance Programme – Pre-school (27-30 month review) | 293,081 | 13,956 | 12,624 | 90.5 | 12,265 | 87.9 | 2,286 | 18.6 |
|  | Child exposed to second hand smoke at 27–30months |  | 293,081 | 13,956 | 12,624 | 90.5 | 12,190 | 87.3 | 1,279 | 10.5 |
| Breastfeeding | Exclusive breast milk feeding at 6–8weeks | Child Health Surveillance Programme – Pre-school (6-8 week review) | 293,081 | 13,956 | 13,062 | 93.6 | 12,876 | 92.2 | 3,453 | 26.8 |
|  | Any breast milk feeding at 6–8weeks |  | 293,081 | 13,956 | 13,062 | 93.6 | 12,876 | 92.2 | 4,817 | 37.4 |
| Immunisations | Complete coverage of universal primary and end infancy immunisations by second birthday^3^ | Scottish Immunisation and Recall System (SIRS) | 296,817 | 14,134 | N/A | N/A | N/A | Assumed 100 | 13,239 | 93.6 |
| Dental attendance | Any attendance at dentist by second birthday^4^ | Management Information & Dental Accounting System (MIDAS) | 293,081 | 13,956 | N/A | N/A | N/A | Assumed 100 | 9,366 | 67.1 |
| Developmental concerns | Any developmental concern at 27–30months | Child Health Surveillance Programme – Pre-school (27-30 month review) | 293,081 | 13,956 | 12,624 | 90.5 | 10,251 | 73.5 | 2,259 | 17.9 |
|  | Any concern about speech, language and communication development at 27–30months |  | 293,081 | 13,956 | 12,624 | 90.5 | 12,044 | 86.3 | 1,579 | 12.5 |
|  | Any concern about social and emotional development at 27–30months^5^ |  | 293,081 | 13,956 | 12,624 | 90.5 | 11,983 | 85.9 | 1,035 | 8.2 |
| Overweight and obesity | Child at risk of overweight or obesity (BMI ≥85th centile) at 27–30 months^6^ | Child Health Surveillance Programme – Pre-school (27-30 month review) | 293,081 | 13,956 | 12,624 | 90.5 | 8,816 | 63.2 | 3,554 | 40.3 |
|  | Child clinically obese (BMI ≥98th centile) at 27–30months |  | 293,081 | 13,956 | 12,624 | 90.5 | 8,816 | 63.2 | 1,052 | 11.9 |
| Accidents and injuries | Any hospital admission for unintentional injury by third birthday^7^ | SMR01 – Hospital Admissions data | 293,081 | 13,956 | N/A | N/A | N/A | Assumed 100 | 479 | 3.4 |
|  | Any hospital admission for unintentional poisoning, burn or scald by third birthday^8^ |  | 293,081 | 13,956 | N/A | N/A | N/A | Assumed 100 | 141 | 1.0 |
|  | Any hospital admission for unintentional long bone fracture or head injury by third birthday^9^ |  | 293,081 | 13,956 | N/A | N/A | N/A | Assumed 100 | 252 | 1.8 |
| Child protection interventions | Placed on child protection register at any point between birth and third birthday | Scottish Government | 297,337 | 14,159 | N/A | N/A | N/A | Assumed 100 | 385 | 2.7 |
|  | Placed on child protection register for ≥6months between birth and third birthday |  | 297,337 | 14,159 | N/A | N/A | N/A | Assumed 100 | 179 | 1.3 |
|  | ‘Looked After Child’ status at any point between birth and third birthday |  | 297,337 | N/A | N/A | N/A | N/A | Assumed 100 | 292 | 2.1 |
|  | ‘Looked After Child’ status for ≥6months between birth and third birthday |  | 297,337 | N/A | N/A | N/A | N/A | Assumed 100 | 256 | 1.8 |

**Notes**

1 Total number in cohort varies slightly depending on the data source. Because data are not linked together, we are unable to establish the ‘true’ number. We therefore display the cohort number according to the data source in question.

2 Data are released for four quarters within each year. In order to give an average, we present data as a mean for the quarter.

3 For the purposes of the UHVP evaluation, 'complete coverage of universal primary and end infancy immunisations by 2nd birthday' is defined as the child having received:

3 doses of the 5 in 1 (DOB up to end July 2017) or 6 in 1 (DOB from Aug 2017) vaccine

1 dose of pneumococcal vaccine (1 provided at ≥12 months)

1 dose of HiB/MenC booster (provided at ≥12 months)

1 dose of MMR (provided at ≥12 months)

4 Attendance at a dentist includes attendance at any NHS dentist providing general dental services

Independent contractor 'high street' dentists and the Public Dental Service are both included

5 A concern about a child's social or emotional development is defined as a concern being recorded against:

The social, emotional, behavioural, or attention domains (reviews provided up to end March 2017)

The personal/social or emotional/behavioural (reviews provided from April 2017)

6 A high % of 27-30 month review records do not provide valid data on the child's BMI

The % varies moderately between health boards (from around 40% to 10%, ie 60% to 90% completeness)

This has been the case fairly consistently from introduction of the 27-30 month review in 2013 to the present

Among children with BMI data available, the % found to be overweight or obese at 27-30 months is very high

This may reflect use of the WHO growth standard (rather than UK90 growth reference) for this age group, and possibly selective weighing of heavier children

7 SMR01 emergency admission type 32, 33, 34, 35 (indicating injury admission) and following ICD10 codes against main or other condition

S00-T78 (any injury or poisoning) + V01-V99 or W00-X59 (accidental injury)

8 SMR01 emergency admission type 32, 33, 34, 35 (indicating injury admission) and following ICD10 codes against main or other condition

T36-T50 (poisoning due to drugs) or T51-T65 (poisoning due to other substances) + X40-49 (unintentional poisoning)

T20-T32 (burns [including electrical] and scalds) + W85-W99 or X00-X19 (unintentional burns and scalds)

9 SMR01 emergency admission type 32, 33, 34, 35 (indicating injury admission) and following ICD10 codes against main or other condition

S00-S09 (head injury), S42 (#upper arm), S52 (forearm), S72 (femur), S82 (lower leg) or any of: T02.2, T02.3, T02.4, T02.5, T02.6, T10, T12 (multiple/unspecified limb)

+ V01-V99 or W00-W64 (accidental injury) or X58-X59 (accidental exposure)
